# Surgical Explantation of an Amplatzer Device for Patent Foramen Ovale Closure in a Patient With Nickel Allergy: A Case Report

**DOI:** 10.1002/ccr3.70006

**Published:** 2024-12-15

**Authors:** Ujjawal Kumar, Usman Aslam, Anthony Cooper, Zain Khalpey

**Affiliations:** ^1^ School of Clinical Medicine University of Cambridge Cambridge UK; ^2^ Department of Cardiothoracic Surgery HonorHealth Scottsdale Arizona USA

**Keywords:** atrial septal defect, closure device, nickel allergy, nickel hypersensitivity, occluding devices, patent foramen ovale

## Abstract

Patent foramen ovale (PFO) closure using percutaneous devices, such as the Amplatzer occluder, is a common treatment for patients with a history of cryptogenic stroke or transient ischemic attack (TIA). Although generally well‐tolerated, some patients may develop adverse reactions to the device materials, particularly in the presence of a nickel allergy. Symptoms can include chest pain, rashes, and migraines, which may necessitate surgical removal of the device. In such cases, careful surgical planning and execution are essential to ensure successful outcomes and symptom resolution. We present the case of a 39‐year‐old female with existing PFO closure using an Amplatzer device, who developed severe, persistent atypical chest pain radiating to her upper extremities and occasional migraines 1 month after Amplatzer implantation. Patch testing confirmed a nickel allergy, prompting a referral to our service for surgical explantation of the device. The patient underwent Amplatzer device removal and reconstruction of the interatrial septum via sternotomy. The surgical technique involved meticulous excision of the device while preserving maximal septal tissue, allowing for primary closure of the interatrial septum without the need for a pericardial patch. Intraoperatively, significant scar tissue and inflammation were observed surrounding the device and atrial septal tissue, necessitating careful excision while preserving healthy tissue. The left atrial appendage was oversewn using a polypropylene suture for thromboembolism prophylaxis. Amniotic membrane allograft and autologous platelet‐rich plasma were applied to promote wound healing, with specialized suture tapes used for chest closure to avoid using stainless steel sternal wires (the most common alloy, 316 L surgical steel contains around 15% nickel) and minimize the risk of sternal complications. The patient tolerated the procedure well, with complete resolution of symptoms following device removal. At follow‐up, she reported improved exercise tolerance, enhanced quality of life, and was able to discontinue her pregabalin medication after 30 months of use (600 mg once daily). She returned to work with light duties 6 weeks postoperatively. This case highlights the importance of preoperative allergy testing to consider nickel allergy in patients before implanting devices such as an Amplatzer, as well as after considering de novo nickel sensitization as a potential cause when such symptoms develop after implantation. This case also highlights the value of surgical intervention in alleviating symptoms and optimizing patient outcomes in cases of device‐related complications.

## Graphical abstract

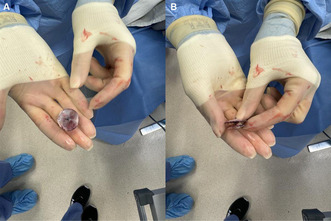

**Summary**

- Preoperative nickel allergy testing should be considered before implanting Amplatzer devices to prevent hypersensitivity reactions.
- If symptoms develop, surgical removal can be safely achieved with careful planning.
- Further research is needed to evaluate patient‐specific risk factors and the overall prevalence of such hypersensitivity reactions.

## 1 Introduction

**FIGURE 1:**
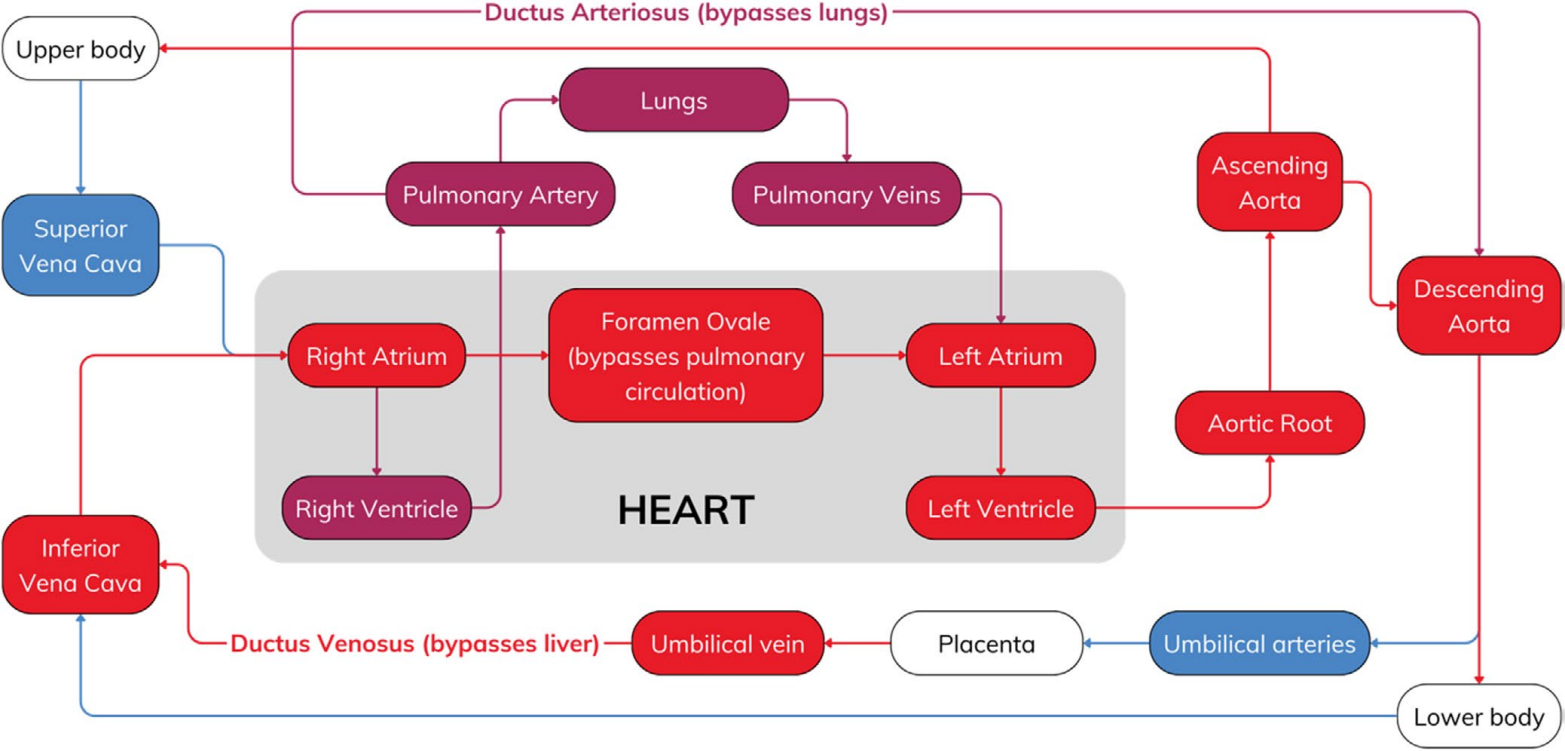
The fetal circulation, showing the key role of the foramen ovale as an intracardiac right–to‐left shunt, bypassing the pulmonary circulation.

A patent foramen ovale (PFO) is a congenital heart defect characterized by a persistent opening between the left atrium (LA) and the right atrium (RA). In utero, the foramen ovale (FO) plays a crucial role in allowing oxygenated blood from the placenta (low systemic vascular resistance) to bypass the pulmonary circulation (liquid‐filled lungs, high pulmonary vascular resistance) and directly enter the systemic circulation (Figure 1) [1]. The foramen ovale typically closes in the first few months of life, but this closure is incomplete in approximately 25% of people, resulting in a PFO [2]. Though most individuals remain asymptomatic, a PFO can allow paradoxical embolism, where a venous thrombus passes from the RA to the LA via the PFO, and onwards to the brain [3, 4]. This can result in a cryptogenic stroke, which has no clear etiology despite investigation, accounting for between 25% and 40% of all ischemic strokes [5].

Various devices have been developed to reduce the stroke risk associated with PFOs, such as the Amplatzer PFO Occluder (Abbott Laboratories, Chicago, IL). It is a self‐expanding double saucer‐shaped device made of a Nitinol mesh (55% nickel and 45% titanium) with a central connecting cylinder. It is implanted percutaneously [6] and has shown promise in clinical trials [7, 8, 9], reducing the risk of recurrent stroke in PFO patients [10]. However, some potential disadvantages are its relatively large bulk, the capacity for incomplete endothelialization with thrombus formation, and the theoretical risk of nickel toxicity due to the nitinol material [11]. Symptoms of nickel allergy include skin rash, itching, redness, and swelling at the implant site [12]. Severe systemic reactions, such as fever, dyspnea, and pericardial effusion, are rare but recognized complications [13]. They typically occur as part of systemic nickel allergy syndrome, a severe form of nickel allergy, estimated to have an incidence of around 5% [14].

We present a case where a PFO was an incidental finding following a transient ischemic attack (TIA). The patient underwent PFO device closure but subsequently developed nickel hypersensitivity symptoms, which resolved following surgical explantation of the Amplatzer. This case highlights the importance of considering nickel allergy when using nitinol‐containing medical implants and the need for close post‐procedure monitoring.

## 2 Case History

A 39‐year‐old woman with a history of transient ischemic attack (TIA) was seen in January 2024 at our center for surgical evaluation. After experiencing a TIA in July 2021, transesophageal echocardiography (TEE) revealed a PFO. In August 2021, she underwent percutaneous PFO closure with an Amplatzer device. Around a month later (September 2021), she developed occasional migraines and constant atypical chest pain radiating to her bilateral upper extremities, present at rest and with exertion. Pregabalin (600 mg once daily) was commenced for symptom control in October 2021. The patient had no other medical history and did not report any known allergies prior to Amplatzer implantation.

## 3 Differential Diagnosis, Investigations, and Treatment

To investigate the cause of the chest pain and aid in surgical planning, diagnostic tests were performed. Coronary angiography was unremarkable, with CT angiography demonstrating normal aortic and branch vessel anatomy. Echocardiography was largely normal other than significant inflammation visualized in the interatrial septum. Patch testing was conducted using the North American Baseline Series (NA‐1000) patch testing protocol, which tests for nickel, alongside a wide range of other allergens [15]. Additionally, titanium patch testing was carried out, as this is the other metal in nitinol. Hapten patches were applied to the patient's back and left in place for 48 h, after which they were removed and skin evaluated for any reactions. A second reading was undertaken at 96 h to evaluate any delayed reaction. A strong (3+) reaction was observed at 48‐h follow‐up, with pruritic papules being seen exclusively on the nickel testing site. The reaction was graded using the International Contact Dermatitis Research Group guidelines (Table 1) [16]. The lack of a reaction to any of the other testing sites, including titanium, confirmed that the patient had no other allergies or sensitivities.

**TABLE 1:** International Contact Dermatitis Research Group (ICDRG) criteria for grading patch test results.

Symbol	Morphology	Assessment
−	No reaction	Negative reaction
?+	Faint erythema only	Doubtful reaction
+	Erythema, infiltration, possibly papules	Weak positive reaction
++	Erythema, infiltration, papules, vesicles	Strong positive reaction
+++	Intense erythema, infiltrate, coalescing vesicles	Extreme positive reaction
IR	Various morphologies, e.g., soap effect, bulla, necrosis	Irritant reaction

Upon presentation to our center, the patient's BMI was 35.2 kg/m^2^, with risk scores and laboratory results as shown in Table 2. Since referral to our center, her C‐reactive protein (CRP) levels had risen, indicating chronic inflammation that presents a high cardiovascular risk, warranting intervention. Non‐surgical treatment options discussed with the patient included a range of pharmacological therapies:

1. Medications typically used for neuropathic pain:
  - Increasing the dose of pregabalin.
  - Trialing a tricyclic antidepressant such as amitriptyline.
2. Addition of steroids or alternative immunomodulatory medications.

**TABLE 2:** Clinical risk scores and laboratory test results that were considered as part of the clinical evaluation of this patient.

Risk Score/Test	Result
CHA_2_DS_2_‐VASc	3
HAS‐BLED	1
Thakar AKI risk score	1.8%
C‐reactive protein (pre‐referral)	8.7 mg/L
C‐reactive protein (when seen in clinic)	12.0 mg/L

**FIGURE 2:**
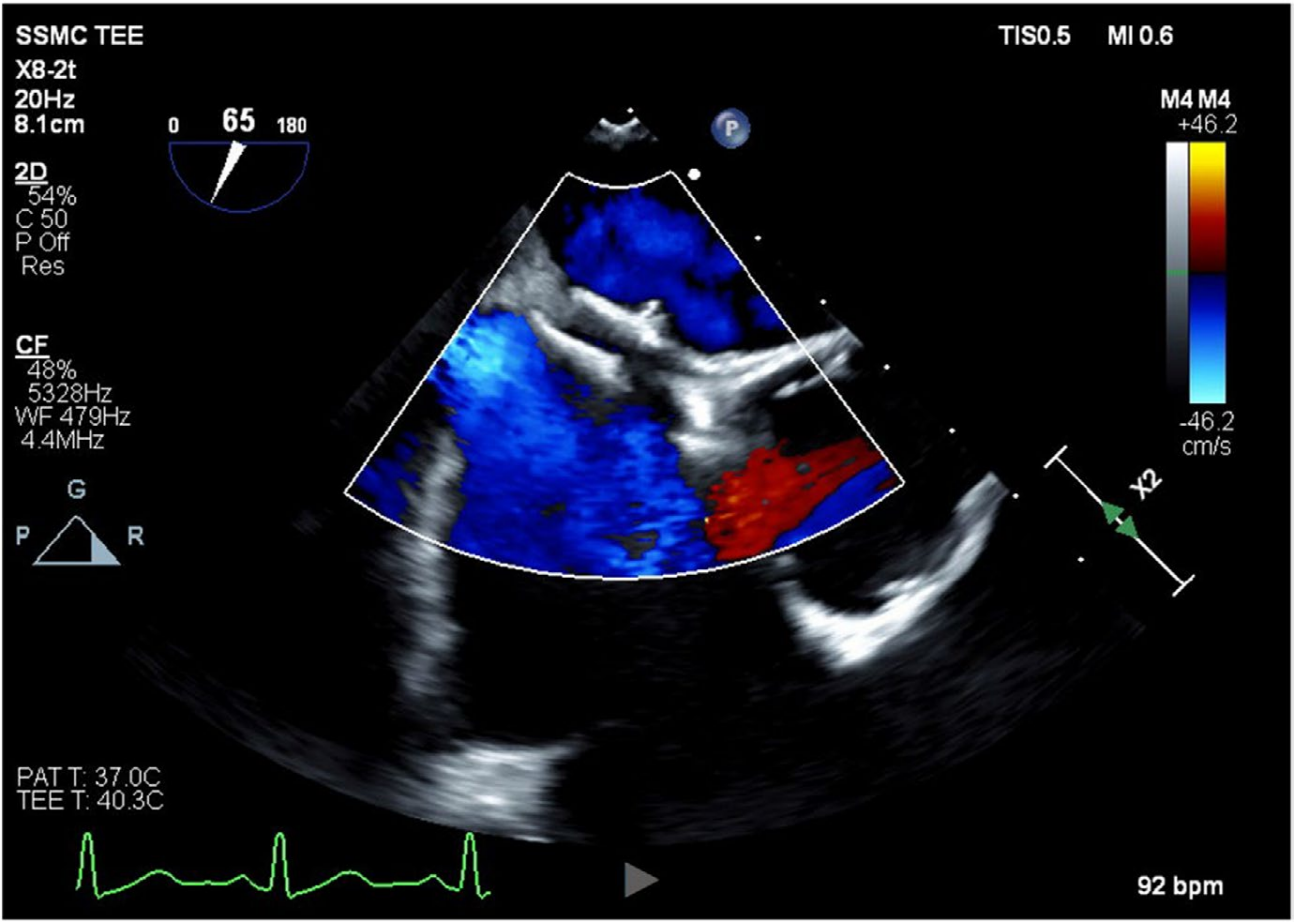
Intraoperative transesophageal echocardiography prior to atriotomy showed complete closure of the patent foramen ovale, with a nickel‐based Amplatzer occlusion device in situ.

**FIGURE 3:**
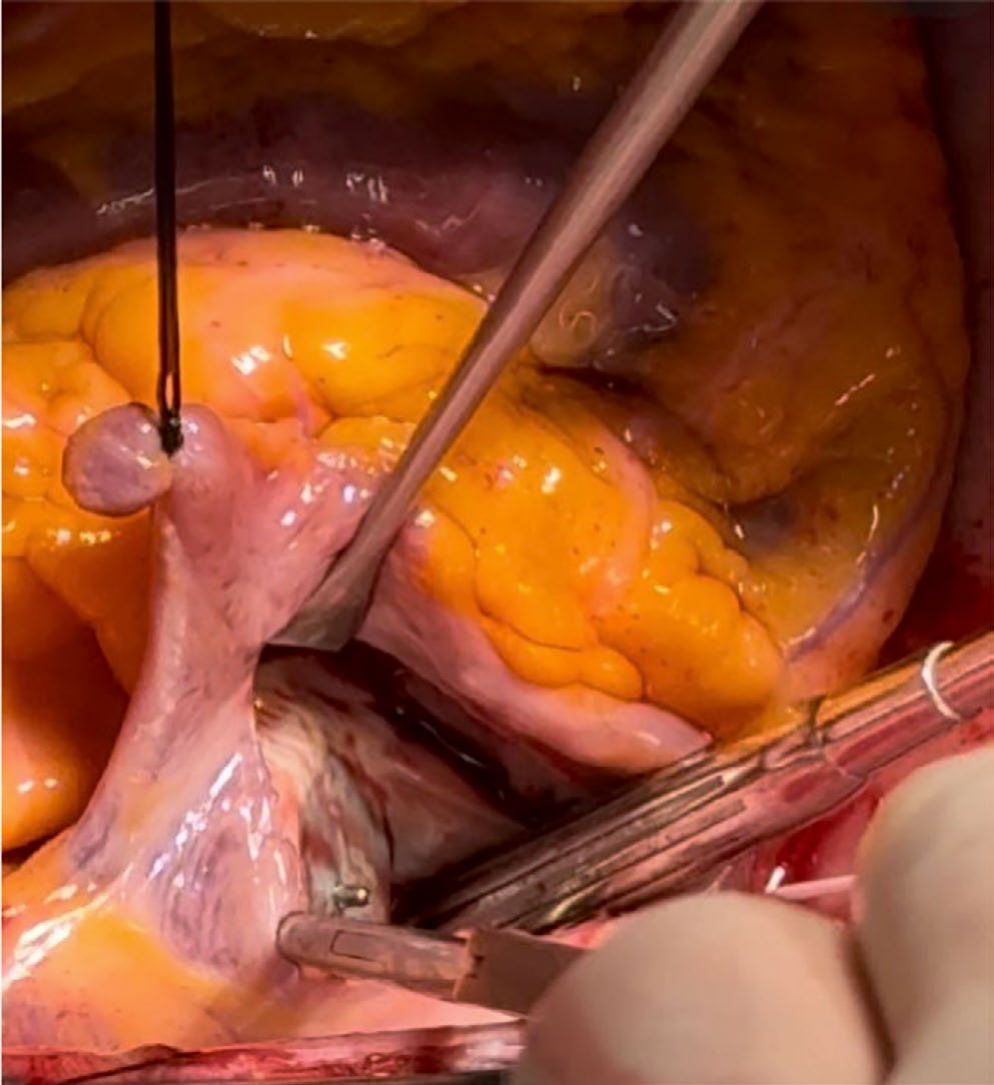
Following right atriotomy for visualization and surgical access, the interatrial septum was found to be significantly inflamed.

After a discussion of treatment options, the decision was made to surgically explant the Amplatzer via a full median sternotomy, reconstruct the interatrial septum, and exclude the left atrial appendage for stroke risk reduction. Cardiopulmonary bypass was initiated using percutaneous bicaval venous (25Fr) and aortic (20Fr) cannulae and 1 L of Del Nido's cold blood cardioplegia (4:1) was given antegrade to achieve electromechanical arrest for myocardial protection. TEE confirmed complete PFO closure by the Amplatzer device in situ (Figure 2). Following right atriotomy, dense adhesions were encountered around the Amplatzer device, and the septal tissue was noted to be heavily inflamed and scarred (Figure 3). This presented a significant challenge, which required a few specific strategies to facilitate Amplatzer explantation. Surrounding structures were gently retracted by the first assistant to avoid putting undue tension on the septum and device. Frequent irrigation was used to maintain visualization, and there was close communication with the anesthesia team to ensure no repositioning of the patient during critical portions of the procedure.

The Amplatzer was meticulously excised using an #11 blade, taking care to preserve the maximum amount of septal tissue and eliminating the need for a pericardial patch. Next, the left atrial appendage was oversewn via a transseptal approach, followed by the interatrial septum, both in two layers with a running 4–0 polypropylene suture (Prolene, Ethicon Inc., Cincinnati, OH).

**FIGURE 4:**
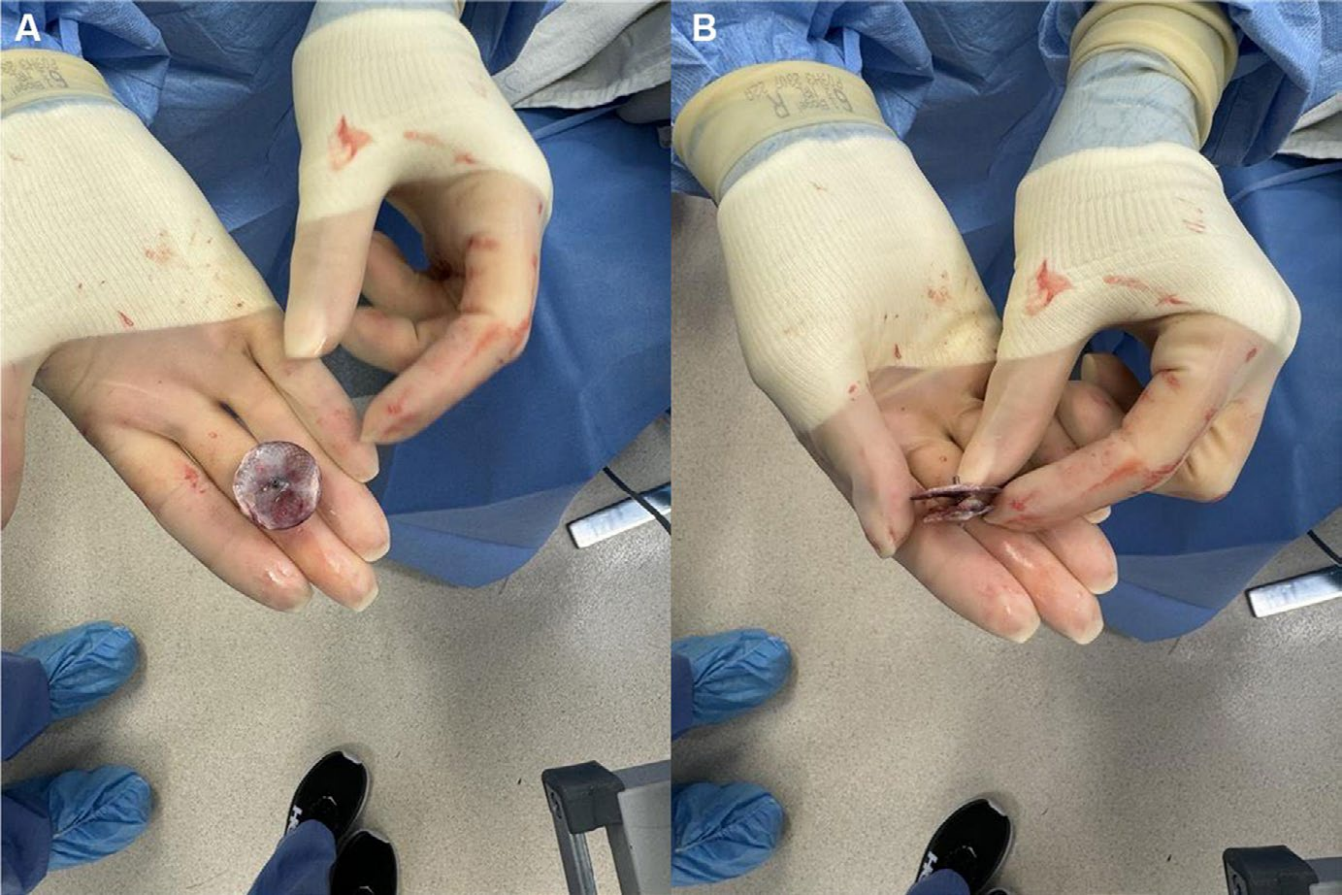
The explanted Amplatzer device as seen (A) from above, and (B) in cross‐section. The device was sent for pathology and culture as is standard in our practice for any explanted implants or specimens.

The patient was weaned from cardiopulmonary bypass (cardiopulmonary bypass time of 62 min and aortic cross‐clamp time of 47 min) and decannulated without complication. TEE confirmed acceptable septal reconstruction with no residual septal defect and complete left atrial appendage closure. The pericardium was approximated using three silk sutures, and bilateral pleural and mediastinal chest tubes were placed. Sternal closure was performed using suture tapes (TigerTape & FiberTape, Arthrex Inc., Naples, FL) instead of conventional steel wires given her nickel allergy. The suture tape system also provides additional strength compared to steel wires [17], which was particularly important given that her BMI of 35.2 kg/m^2^ puts her at high risk of postoperative sternal complications [18]. This patient's chronically raised CRP indicated a systemic pro‐inflammatory state, which would further impair wound healing. As previously discussed for patients at high risk of sternal complications [19, 20], 160 mg of human amniotic membrane allograft (Salera Mini Membrane, MTF Biologics, Edison, NJ, USA) was placed over the sternum and superficial fascia, and autologous platelet‐rich plasma (PRP) was applied to the wound before skin closure. These adjuncts promote wound healing through their anti‐inflammatory cytokines and growth factors. The explanted Amplatzer device (Figure 4) was sent for pathology and culture, which showed no signs of bacterial growth.

## 4 Outcome and Follow‐Up

The patient was transferred to the ICU in stable condition, extubated 6 h later, and began mobilizing soon after. The patient reported that her symptoms had resolved by the next day. Postoperative analgesia was achieved using minimal opioids, with the patient receiving 90 mg of dronabinol (a synthetic cannabinoid) perioperatively to reduce opioid requirement [21]. She was discharged home on the fifth postoperative day after an uneventful hospital admission.

At her 2‐week, 3‐ and 6‐month follow‐up appointments, her sternal incision was healing well with no evidence of dehiscence or infection. She reports complete symptom resolution and significantly enhanced quality of life. She returned to work with light duties 6 weeks postoperatively. She no longer requires her pregabalin medication (600 mg once daily), which was discontinued in April 2024 after around 30 months of use (October 2021 to March 2024). Her exercise tolerance has increased from barely being able to walk 100 m preoperatively due to chest pain and dyspnea, to now walking at least 3 km per day and engaging in cardiac rehabilitation. Additionally, despite her high risk of sternal complications, she experienced no sternal complications, and her sternum was stable at follow‐up. Further long‐term follow‐up is planned to monitor her progress every 6 months, with regular screening for symptoms, as well as annual echocardiography to monitor cardiac function and structure, ensuring maintained PFO closure.

## 5 Discussion

This case highlights key considerations in managing patients with nickel allergy and complications of septal occluder devices. While these devices are generally safe and effective, their nickel component can trigger allergic reactions, leading to persistent symptoms and potentially necessitating device removal [13]. Clinicians should maintain a high index of suspicion for nickel allergy in patients with persistent symptoms following Amplatzer implantation. These may include ongoing chest pain, rashes, migraines, or other atypical presentations, as demonstrated in this case.

The patient's nickel allergy was crucial in her post‐PFO closure symptoms. The device's high nickel content likely triggered a chronic inflammatory response, evidenced by elevated CRP levels and significant inflammation observed intraoperatively. The recognized rise in serum nickel concentrations within 24 h of Amplatzer implantation [22, 23] may explain the rapid symptom onset. This case highlights the importance of considering allergies when selecting intracardiac devices and closely monitoring for post‐implantation allergic reactions.

### 5.1 Importance of Preoperative Screening

This patient was unaware of any nickel allergy at the time of Amplatzer implantation. There was therefore no perceived contraindication to using a nitinol‐based device at that time. Routine preoperative nickel sensitivity testing is not standard practice at our institution or others. Consequently, the nickel allergy was only diagnosed through postoperative patch testing to investigate her symptoms. While some studies [24, 25] suggest limited value in pre‐procedure testing, this case highlights the potential benefits of routine preoperative screening in patients undergoing cardiac device implantation. Preoperative knowledge of the allergy would improve informed consent. Only with full awareness of potential sensitivities can patients and clinicians make proper informed decisions.

Percutaneous closure is often favored to avoid invasive surgery, cardiopulmonary bypass, a lengthier recovery, and potentially higher procedural risks. However, all currently approved ASD and PFO closure devices contain nitinol. In cases of severe nickel allergy, direct surgical closure may therefore be more appropriate. If surgery is not feasible, devices with less nitinol content, such as the GORE CARDIOFORM Septal Occluder (W.L. Gore and Associates Inc., Flagstaff, AZ) should be considered. This exhibits in vitro nickel elution levels similar to a placebo and significantly lower levels than the Amplatzer [26].

While the prevalence of nickel allergy in patients with implanted cardiac devices is difficult to quantify due to the relative rarity of nickel hypersensitivity, multiple case reports discuss similar presentations following implantation of nickel‐containing cardiac devices [12, 13] such as the Amplatzer [27]. These reports consistently recommend surgical explantation to resolve symptoms, as well as alternative methods of sternal closure methods to avoid steel sternal wires due to their nickel content, a strategy that we employed in this case. A systematic review and meta‐analysis of endovascular devices and nickel hypersensitivity found a significant association with adverse outcomes following implantation of a nickel‐containing endovascular device in patients with a patch‐test confirmed nickel allergy [28].

### 5.2 Surgical Management and Techniques

Intraoperatively, the heavily scarred and inflamed septal tissue increased the difficulty of the dissection. However, careful excision while preserving adequate septal integrity facilitated closure without a patch. The left atrial appendage was oversewn for stroke prevention given her high stroke risk (CHA_2_DS_2_‐VASc score of 3), providing an alternative to long‐term anticoagulation. We have previously shown that closing the left atrial appendage allows for the safe elimination of formal anticoagulation, despite postoperative atrial fibrillation [29], improving patient quality of life.

As part of our standard institutional practice, an advanced sternal closure system (suture tapes) was used instead of steel wires to minimize the risk of sternal complications and potential hypersensitivity reactions. These enhance sternal stability, distributing forces evenly and reducing the risk of postoperative sternal dehiscence and mediastinitis. Ex vivo data shows the superiority of this technology compared to steel sternal wires [30], and forthcoming work from our group will highlight our excellent outcomes, surpassing those of steel wires. Sternal wires are commonly made of 316 L surgical steel, which has a nickel content of around 15% [31], and similar hypersensitivity‐type symptoms have previously been reported in response to sternal wires [32]. Therefore, there is a chance that the nickel content in surgical steel may prevent complete symptom resolution if wires had been used. Furthermore, amniotic membrane allograft and autologous platelet‐rich plasma were applied to promote soft tissue healing. They contain growth factors and cytokines that stimulate angiogenesis, reducing inflammation and scar formation [19, 33, 34, 35], which are particularly beneficial in this patient given that her BMI and systemic pro‐inflammatory state (shown by chronically raised CRP) increase her risk of sternal complications. Their use was effective, as shown by the absence of sternal complications, despite her high risk. While these closure techniques do add costs compared to standard steel wires, our institutional experience has shown this approach to significantly reduce sternal complication rates (wound infection, dehiscence, extended hospital admissions, readmissions), all of which this patient was at high risk for. These complications would incur costs far in excess of the costs of this enhanced sternal closure protocol, thus it was felt to be worth it in this case.

### 5.3 Outcomes and Implications

The rapid symptom resolution following device removal highlights the efficacy of surgical intervention in managing nickel hypersensitivity symptoms and improving quality of life. This case highlights the importance of considering nickel allergies when selecting intracardiac devices and maintaining vigilance for allergic reactions post‐implantation. The use of an advanced sternal closure system (ultrahigh molecular weight polyethylene suture tapes) and biologic adjuncts (amniotic membrane and platelet‐rich plasma) may help mitigate wound healing complications in similar high‐risk patients [36, 37]. Ongoing research and technological advancements may continue to refine management strategies for these challenging clinical scenarios.

## 6 Conclusions

Nickel allergy is an important consideration in patients with persistent symptoms after placement of nitinol devices such as an Amplatzer. Surgical removal, while technically challenging, can be achieved safely with careful planning and appropriate preoperative planning. We advocate for preoperative allergy testing where feasible, before device implantation, to prevent the need for reintervention in the future if hypersensitivity symptoms do occur and allow for better patient informed consent. Further research is needed to evaluate patient‐specific risk factors in developing these symptoms, as well as to identify the overall prevalence of such symptoms.

## Author Contributions

**Ujjawal Kumar:** data curation, formal analysis, investigation, methodology, project administration, writing – original draft, writing – review and editing. **Usman Aslam:** data curation, writing – review and editing. **Anthony Cooper:** data curation, writing – review and editing. **Zain Khalpey:** conceptualization, data curation, project administration, supervision, writing – review and editing.

## Ethics Statement

This case report was exempt from IRB approval requirements subject to patient consent.

## Consent

Written informed consent was obtained from the patient to publish this report in accordance with the journal's patient consent policy.

## Conflicts of Interest

All authors declare no conflicts of interest.

## Data Availability

The relevant data for this case report are available upon reasonable request. The data are not publicly available due to privacy and ethical restrictions.

## References

[1] P. T. Hagen , D. G. Scholz , and W. D. Edwards , “Incidence and Size of Patent Foramen Ovale During the First 10 Decades of Life: An Autopsy Study of 965 Normal Hearts,” Mayo Clinic Proceedings 59 (1984): 17–20, 10.1016/s0025-6196(12)60336-x. 6694427

[2] I. Meissner , J. P. Whisnant , B. K. Khandheria , et al., “Prevalence of Potential Risk Factors for Stroke Assessed by Transesophageal Echocardiography and Carotid Ultrasonography: The SPARC Study,” Mayo Clinic Proceedings 74 (1999): 862–869, 10.4065/74.9.862. 10488786

[3] P. Lechat , J. L. Mas , G. Lascault , et al., “Prevalence of Patent Foramen Ovale in Patients With Stroke,” New England Journal of Medicine 318 (1988): 1148–1152, 10.1056/nejm198805053181802. 3362165

[4] D. E. Thaler , R. Ruthazer , C. Weimar , et al., “Recurrent Stroke Predictors Differ in Medically Treated Patients With Pathogenic vs Other PFOs,” Neurology 83 (2014): 221–226, 10.1212/WNL.0000000000000589. 24928123 PMC4117365

[5] J. L. Saver , “Cryptogenic Stroke,” New England Journal of Medicine 374 (2016): 2065–2074, 10.1056/NEJMcp1503946. 27223148

[6] R. Thaman , G. Faganello , J. R. Gimeno , et al., “Efficacy of Percutaneous Closure of Patent Foramen Ovale: Comparison Among Three Commonly Used Occluders,” Heart 97 (2011): 394–399, 10.1136/hrt.2010.203950. 21296783

[7] K. C. Chan , M. J. Godman , K. Walsh , N. Wilson , A. Redington , and J. L. Gibbs , “Transcatheter Closure of Atrial Septal Defect and Interatrial Communications With a New Self Expanding Nitinol Double Disc Device (Amplatzer Septal Occluder): Multicentre UK Experience,” Heart 82 (1999): 300–306, 10.1136/hrt.82.3.300. 10455079 PMC1729188

[8] L. Søndergaard , S. E. Kasner , J. F. Rhodes , et al., “Patent Foramen Ovale Closure or Antiplatelet Therapy for Cryptogenic Stroke,” New England Journal of Medicine 377 (2017): 1033–1042, 10.1056/nejmoa1707404. 28902580

[9] D. Hildick‐Smith , T. Williams , P. MacCarthy , et al., “Occlutech Percutaneous Patent Foramen Ovale Closure: Safety and Efficacy Registry (OPPOSE),” International Journal of Cardiology 245 (2017): 99–104, 10.1016/j.ijcard.2017.07.058. 28778466

[10] V. Nagaraja , J. Raval , G. D. Eslick , D. Burgess , and A. R. Denniss , “Is Transcatheter Closure Better Than Medical Therapy for Cryptogenic Stroke With Patent Foramen Ovale? A Meta‐Analysis of Randomised Trials,” Heart, Lung & Circulation 22 (2013): 903–909, 10.1016/j.hlc.2013.07.022. 24035325

[11] F. W. Sunderman , “A Review of the Metabolism and Toxicology of Nickel,” Annals of Clinical and Laboratory Science 7 (1977): 377–398. 332046

[12] K. Fukahara , K. Minami , N. Reiss , D. Fassbender , and R. Koerfer , “Systemic Allergic Reaction to the Percutaneous Patent Foramen Ovale Occluder,” Journal of Thoracic and Cardiovascular Surgery 125 (2003): 213–214, 10.1067/mtc.2003.125. 12539013

[13] U. K. Dasika , K. R. Kanter , and R. Vincent , “Nickel Allergy to the Percutaneous Patent Foramen Ovale Occluder and Subsequent Systemic Nickel Allergy,” Journal of Thoracic and Cardiovascular Surgery 126 (2003): 2112, 10.1016/j.jtcvs.2003.06.005. 14688749

[14] L. Ricciardi , A. Arena , E. Arena , et al., “Systemic Nickel Allergy Syndrome: Epidemiological Data From Four Italian Allergy Units,” International Journal of Immunopathology and Pharmacology 27 (2014): 131–136, 10.1177/039463201402700118. 24674689

[15] P. C. Schalock , C. A. Dunnick , S. Nedorost , et al., “American Contact Dermatitis Society Core Allergen Series: 2020 Update,” Dermatitis 31 (2020): 279–282, 10.1097/DER.0000000000000621. 32947457

[16] J. D. Johansen , K. Aalto‐Korte , T. Agner , et al., “European Society of Contact Dermatitis Guideline for Diagnostic Patch Testing—Recommendations on Best Practice,” Contact Dermatitis 73 (2015): 195–221, 10.1111/cod.12432. 26179009

[17] Arthrex Inc , “Data on File (APT 5056),” 2021.

[18] C. Schimmer , W. Reents , S. Berneder , et al., “Prevention of Sternal Dehiscence and Infection in High‐Risk Patients: A Prospective Randomized Multicenter Trial,” Annals of Thoracic Surgery 86 (2008): 1897–1904, 10.1016/j.athoracsur.2008.08.071. 19022005

[19] U. Kumar , U. Aslam , and Z. Khalpey , “Sternal Complications Following Coronary Artery Bypass Grafting and Robicsek Repair: Comprehensive Sternal Reconstruction With Sternal Plating and the Use of Novel Biologic Therapies,” Cureus 16 (2024): e59719, 10.7759/cureus.59719. 38841045 PMC11152356

[20] Z. Khalpey , U. Kumar , Z. I. Khalpey , P. Hitscherich , E. Chnari , and M. Long , “Novel Use of an Aseptically Processed Amnion‐Chorion Placental Allograft to Complement Wound Closure in High‐Risk Sternotomy Patients: Clinical Safety and Outcomes,” Cureus (2024), 10.7759/cureus.73322. PMC11626682 39655141

[21] U. Kumar , A. R. Macko , N. Kang , N. G. Darian , F. O. Salek , and Z. Khalpey , “Perioperative Cannabinoids Significantly Reduce Postoperative Opioid Requirements in Patients Undergoing Coronary Artery Bypass Graft Surgery,” Cureus 16 (2024): e58566, 10.7759/cureus.58566. 38765405 PMC11102566

[22] M. W. Ries , C. Kampmann , H.‐J. Rupprecht , G. Hintereder , G. Hafner , and J. Meyer , “Nickel Release After Implantation of the Amplatzer Occluder,” American Heart Journal 145 (2003): 737–741, 10.1067/mhj.2003.7. 12679773

[23] O. Elkiran , C. Karakurt , G. Kocak , and C. Taskapan , “Serum Nickel and Titanium Levels After Transcatheter Closure of Atrial Septal Defects With Amplatzer Septal Occluder,” Cardiology Research and Practice 2019 (2019): 7891746, 10.1155/2019/7891746. 30719342 PMC6334312

[24] I. Kimber and D. A. Basketter , “Allergic Sensitization to Nickel and Implanted Metal Devices: A Perspective,” Dermatitis 33 (2022): 396–404, 10.1097/DER.0000000000000819. 34845168 PMC9674446

[25] A. Bogdanova‐Bennett , A. Sagi , V. Asopa , R. E. Field , and D. H. Sochart , “Nickel Hypersensitivity and Skin Patch Testing in Total Hip Replacement Surgery: A Systematic Review,” EFORT open reviews 6 (2021): 825–838, 10.1302/2058-5241.6.210051. 34760283 PMC8559563

[26] C. D. Resor , A. M. Goldminz , P. Shekar , R. Padera , P. T. O'Gara , and P. B. Shah , “Systemic Allergic Contact Dermatitis due to a GORE CARDIOFORM Septal Occluder Device: A Case Report and Literature Review,” A Case Report and Literature Review 2 (2020): 1867–1871, 10.1016/j.jaccas.2020.05.091. PMC8299130 34317069

[27] M. N. Gritti , G. Mets , A. Jevremovic , and L. N. Benson , “Atrial Septal Defect Devices and Nickel Allergies: An Unexpected Silver Lining,” CJC pediatric and congenital heart disease 2 (2023): 146–149, 10.1016/j.cjcpc.2023.03.002. 37969350 PMC10642119

[28] A. M. Guéroult , A. Al‐Balah , A. H. Davies , and J. Shalhoub , “Nickel Hypersensitivity and Endovascular Devices: A Systematic Review and Meta‐Analysis,” Heart (British Cardiac Society) 108 (2022): 1707–1715, 10.1136/heartjnl-2021-319940. 34702756

[29] Z. Khalpey , U. Aslam , P. Wilson , J. Deckwa , and U. Kumar , “Prophylactic Left Atrial Appendage Ligation During Coronary Artery Bypass Graft Surgery Allows Safe Avoidance of Anticoagulation Regardless of Postoperative Atrial Fibrillation,” Cureus 16 (2024): e59876, 10.7759/cureus.59876. 38854212 PMC11157991

[30] Arthrex Inc , “FiberTape® and TigerTape™,” 2024, https://www.arthrex.com/cardiothoracic‐surgery/fibertape‐sternal‐closure‐system.

[31] C.‐M. Shih , Y.‐Y. Su , S.‐J. Lin , and C.‐C. Shih , “Failure Analysis of Explanted Sternal Wires,” Biomaterials 26 (2005): 2053–2059, 10.1016/j.biomaterials.2004.07.005. 15576179

[32] E. M. Zywicka , T. Theologou , S. Love , and O. Nawaytou , “Sternal Wires–Induced Severe Systemic Inflammatory Response and Cardiac Tamponade,” Annals of Thoracic Surgery 107 (2019): e175–e176, 10.1016/j.athoracsur.2018.06.090. 30266619

[33] G. Castellanos , Á. Bernabé‐García , J. M. Moraleda , and F. J. Nicolás , “Amniotic Membrane Application for the Healing of Chronic Wounds and Ulcers,” Placenta 59 (2017): 146–153, 10.1016/j.placenta.2017.04.005. 28413063

[34] B. L. Eppley , W. S. Pietrzak , and M. Blanton , “Platelet‐Rich Plasma: A Review of Biology and Applications in Plastic Surgery,” Plastic and Reconstructive Surgery 118 (2006): 147e–159e, 10.1097/01.prs.0000239606.92676.cf. 17051095

[35] Z. Khalpey , U. Kumar , Z. I. Khalpey , P. Hitscherich , E. Chnari , and M. Long , “Novel Use of an Aseptically Processed Amnion‐Chorion Placental Allograft to Complement Wound Closure in High‐Risk Sternotomy Patients: Clinical Safety and Outcomes,” Cureus 16 (2024): e73322, 10.7759/cureus.73322. 39655141 PMC11626682

[36] Y. Menchisheva , U. Mirzakulova , and R. Yui , “Use of Platelet‐Rich Plasma to Facilitate Wound Healing,” International Wound Journal 16 (2019): 343–353, 10.1111/iwj.13034. 30440099 PMC7949413

[37] T. Ando , D. Akiyama , H. Okada , H. Furukawa , and M. Takeda , “Novel Sternum Closure Technique Using Ultra High Molecular Weight Polyethylene Sutures,” Kyobu Geka 69 (2016): 1055–1058. 27909272

